# Impact of leadership styles on safety performance in risky workplaces: a meta-analysis

**DOI:** 10.3389/fpsyg.2026.1848283

**Published:** 2026-09-03

**Authors:** Zhihong Li, Jun Tang, Xinya Yang, Jiale Zhang

**Affiliations:** 1 School of Public Administration and Policy, Renmin University of China, Beijing, China; 2 Faculty of Humanities Social Sciences, Nanjing Forestry University, Nanjing, China

**Keywords:** boundary conditions, leadership styles, meta-analysis, risky workplaces, safety performance, theoretical framework

## Abstract

**Introduction:**

Leadership is a key factor in promoting safety performance (whereby organizations or employees strive to minimize workplace injuries and accidents). However, extant findings are controversial and lack a systematic quantitative review of the relationship between different leadership styles and safety performance and its boundary conditions.

**Methods:**

The study constructed a meta-analytic theoretical framework covering 102 studies (107,109 participants) published before 2025 to interpret how the different impacts of leadership styles on safety performance.

**Results:**

The results showed that 13 leadership styles were significantly positively associated with safety performance, and 4 were significantly negatively associated with safety performance. Relative weighted analyses revealed that transformational leadership had the most explainable contribution to safety performance. In addition, moderator analyses indicated that, in some cases, the impacts of leadership styles on safety performance were moderated by cultural factors, power factors, methodological factors, and risk factors.

**Conclusion:**

This study advances the literature by being the first to concurrently integrate 21 distinct leadership styles into a single comparative framework and analyze their fluctuating effects across varying levels of occupational risk, highlighting the importance of different leadership styles in enhancing or inhibiting safety governance.

## 1 Introduction

While work provides numerous benefits, it can also pose physical hazards and risks to employees’ well-being (Arifin et al., 2025; Burgard and Lin, 2013; Durman et al., 2026; Payne et al., 2025; Shukla et al., 2024; Sunstein, 2002). Scenarios such as neglecting safety-related hazards, failing to wear protective equipment, or suffering severe injuries or fatalities in extreme risk workplaces, these scenarios highlight the critical role of workplace safety for both employees and the organizations they serve (Arboh et al., 2024; Payne et al., 2025; Mario and Stephanou, 2017; Suwaidi et al., 2025; Viscusi, 1983; Wang et al., 2023). Despite advancements in occupational safety over recent decades (International Labor Organization, 2024; Swuste et al., 2010), workplace safety issues remain prominent (Beus et al., 2016; Jiang et al., 2024; Takala et al., 2014; Vitale et al., 2024).

Safety performance is the core of workplace safety, referring to the work behaviors of organizations or employees to promote workplace safety, that is, the efforts made by organizations or employees to reduce workplace accidents and injuries (Arifin et al., 2025; Bautista et al., 2024; Kapp, 2012; Neal et al., 2000; Suwaidi et al., 2025). It indicates how well the organization is performing in terms of safety and can be expressed through safety-related events (e.g., accidents and injuries) or safety behaviors (e.g., safety compliance and safety participation; Ali et al., 2020; Mario et al., 2013; Piao and Hahn, 2025), while safety performance broadly encompasses this entire spectrum, the present meta-analysis specifically focuses on the behavioral dimensions—namely, self-reported safety compliance and safety participation—to maintain measurement consistency and avoid the methodological heterogeneity of mixing subjective reports with objective accident rates. However, poor safety performance comes at a high cost to organizations. According to a report by the International Labor Organization, in 2023 alone, there will be approximately 3.5 million deaths globally due to work-related accidents and diseases, with about 240 million occupational accidents and over 200 million victims of work-related illnesses (International Labor Organization, 2024). According to private sector employer reports, there are approximately 2.6 million non-fatal work-related injuries and illnesses (BLS, 2024) within a year. The losses caused by these work-related injuries include loss of life and time (Ajayi et al., 2020; Kim and Park, 2021; Suwaidi et al., 2025), decreased productivity (Galizzi et al., 2023), work fatigue, and poor attitudes (Hadjimanolis et al., 2015; Nyame et al., 2023), which impose a huge burden and pressure on organizations, thereby making workplace safety a top priority for many organizations. Researchers, policy makers, and managers are also working to find out “What factors can improve (or disrupt) workplace safety performance?” (Conchie and Donald, 2006; Hofmann et al., 2017; Payne et al., 2025), and a widely recognized factor is leadership.

Leadership has a critical part in enhancing (or disrupting) safety performance. Some practitioners and policy makers have made significant efforts to reduce accidents and injuries in the workplace, such as establishing policies and practices for reducing workplace risks (Kapp, 2012; Northouse, 2015; Payne et al., 2025; Suwaidi et al., 2025; Vitale et al., 2024). However, a large body of data from researchers has shown inconsistent conclusions, such as that certain categories of positive “constructive” leadership (e.g., empowering leadership, safety leadership, transactional leadership, transformational leadership, and ethical leadership) are found to have a positive, negative, or unrelated relationship with to safety performance and reducing workplace risks (e.g., Beatriz et al., 2014; Gracia et al., 2020; Inness et al., 2010; Quansah et al., 2023; Shafique et al., 2020); and other “negative” leadership behaviors (destructive leadership, humor leadership, narcissistic leadership, abusive leadership, authoritarian leadership, and laissez-faire leadership) are also found to have positive, negative, or unrelated relationships with safety performance (e.g., Jiang and Probst, 2016; Li and Li, 2021; Wei, 2023).

However, to date, while there have been a few studies integrating current empirical evidence of several constructive leadership behaviors (transactional leadership, transformational leadership, and safety leadership) in terms of safety engagement or safety performance (Jiang et al., 2024; Payne et al., 2025), they have only quantitatively or qualitatively integrated the influences of certain leadership styles on safety participation or safety performance, or emphasized the importance of these relationships, without systematically explaining the mechanisms and boundary conditions under which different leadership styles promote or inhibit safety performance. Meta-analytic techniques can compensate for this by not only overcoming the distortion effects of research bias and statistical artifacts caused by study design, sample size, and researchers’ subjective bias, but also improving the validity of statistical analyses, revealing uncertainty in independent studies, discovering differences between general findings and independent studies, establishing cumulative empirical results, providing open and transparent support for reliable evidence, proposing new perspectives, research topics, and directions, and reducing research costs (Barends and Rousseau, 2018; Hunter and Schmidt, 2004; Li et al., 2020; Tang and Li, 2026). While previous few meta-analyses have advanced our understanding by examining isolated leadership behaviors (e.g., transformational or safety leadership), their scope remains largely fragmented. What is genuinely novel in the present study is its comprehensive integration of 21 distinct leadership styles within a single nomological network, allowing for a simultaneous evaluation of their relative weights. Furthermore, this study explicitly contextualizes these relationships by systematically testing boundary conditions, such as cultural norms, power dynamics, and the full continuum of occupational risk levels (spanning high, medium, and low-risk workplaces) that have not been concurrently evaluated in prior reviews. Therefore, determining the impact of leadership styles on safety performance can enable researchers and managers to more accurately weigh the positive and negative consequences that leadership may bring.

Therefore, our study aims to quantitatively integrate the empirical evidence of leadership styles and safety performance. First, we developed a theoretical model and a set of hypotheses about the associations between them. Then, meta-analysis was conducted by screening, coding, and extracting relevant data and using random effects models, researchers can not only identify which leadership styles are associated with safety performance, but also their positive and negative correlations and degree of correlation (i.e., relative weight influence). Without such meta-analysis evidence, our systematic understanding of leadership styles and safety performance in risky workplaces remains limited. Afterwards, we investigated different categories of moderating factors (cultural factors, power factors, methodological factors, and risk factors) that affect their relationship to determine their boundary conditions. Finally, we discussed the significance of these results and provided some suggestions for future research.

## 2 Theoretical background and hypotheses

**Figure 1:**
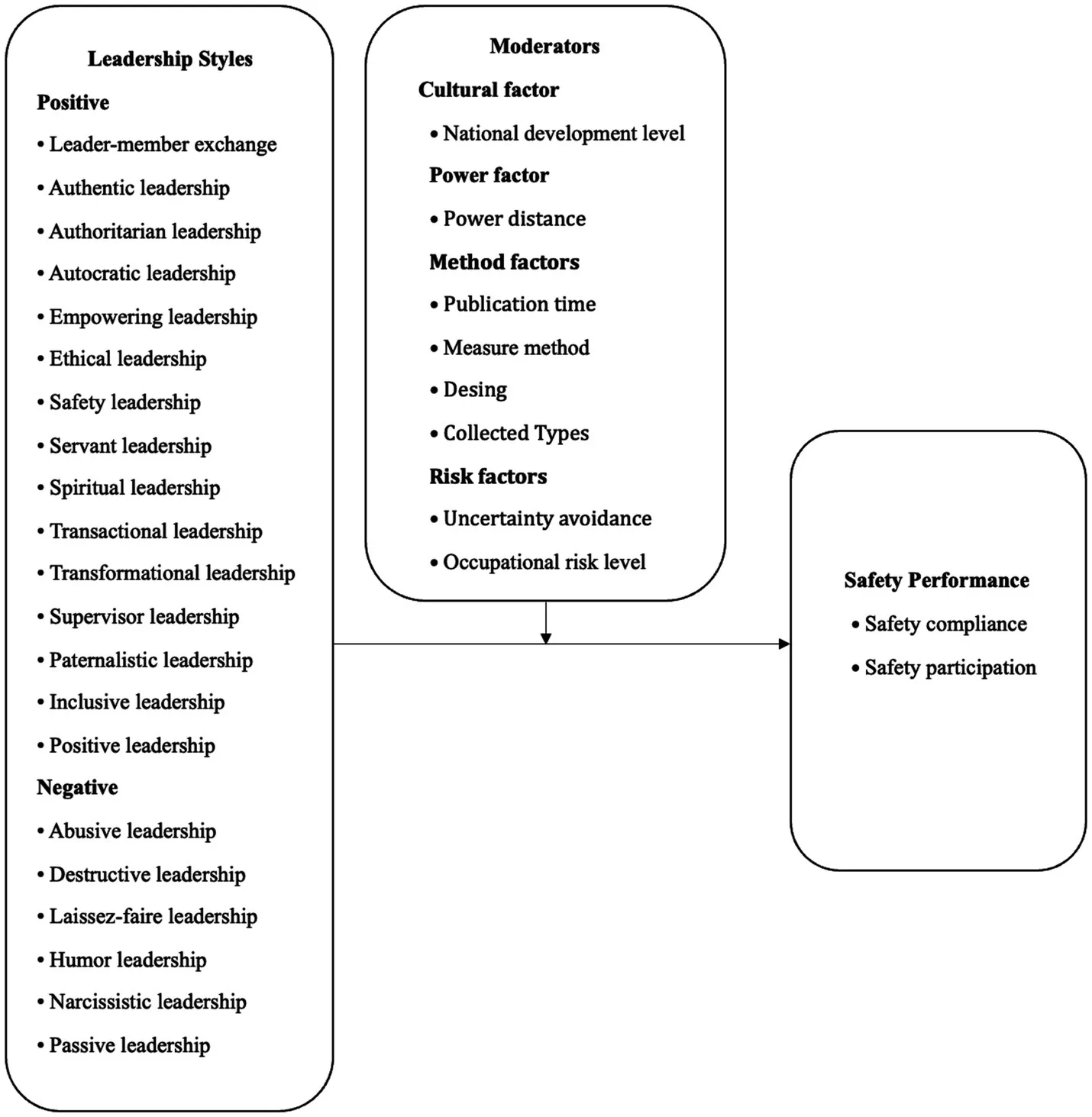
Theoretical research framework.

We constructed a meta-analysis theoretical framework to explain the differences in the influence of various leadership styles on safety performance. Our theoretical framework is shown in Figure 1.

### 2.1 Conceptualizing leadership

Leadership is the ability of an individual to reach a common goal by guiding, motivating, and inspiring others in an organization or team (Bass and Bass, 2008; Durman et al., 2026; Judge et al., 2002; Northouse, 2015; Payne et al., 2025; Suwaidi et al., 2025). It is an essential managerial and organizational skill, acting as a critical element of organizational and team success, and representing a key step in the process by which a leader achieves leadership effectiveness, including mitigating workplace risks and maintaining safety (Clarke and Ward, 2006; Shen et al., 2024). Leadership involves a clear vision and goals, influence, communication skills, teamwork, and innovative thinking. The contribution of leadership is reflected in: (1) enhancing the efficiency and performance of organizations and teams, achieving common goals, (2) establishing a good organizational and team culture, enhancing team cohesion and centripetal force, (3) promoting the personal and career development of employees, improving employee job satisfaction and loyalty, (4) enhancing the innovation ability and competitiveness of organizations and teams, and promoting the development and progress of organizations and teams (Bass and Bass, 2008). Previous studies have shown that there are numerous different styles of leadership, and leadership styles have a significant impact on work outcomes in various research settings, such as employee behavior and performance, work engagement, and organizational commitment, demonstrating that leadership makes a contribution to the success of the organization (Judge et al., 2002; Montano et al., 2017; Zhao et al., 2026). According to the characteristics of leadership styles, they can be divided into transactional, transformational, destructive, servant, ethical, safety, and other leadership styles (see Figure 1 for details).

### 2.2 Conceptualizing safety performance

Safety performance is the work behavior of an organization or employee to promote workplace safety. It is the effort made by an organization or employee to reduce workplace accidents and injuries. It is often used as a dependent variable and measured through a series of safety outcomes (such as accident rates, injuries, etc.), mainly including two dimensions: safety compliance and safety participation (Bautista et al., 2024; Kapp, 2012; Neal et al., 2000). In this study, we operationalize safety performance strictly through these self-reported behavioral dimensions, deliberately excluding objective safety outcomes (such as actual accident or injury rates) to ensure a methodologically homogenous comparison. Safety compliance corresponds to task performance, which refers to the adherence to safety production regulations and operational norms in enterprise production to complete work tasks. It represents the key behaviors that employees will have to adopt in order to keep their workplaces safe, such as following safety procedures, maintaining work procedure standards, and wearing individual protected equipment in the workplace. Beyond that, safety compliance involves employees’ efforts to maintaining a safe workplace through adherence to the organization’s safety-based procedures, rules, and regulations; safety participation corresponds to relationship performance, it refers to actively participating in or engaging in safety production management work outside of work regulations, including actively helping colleagues to do a good job in safety work, actively engage in voluntary safety activities, drills, safety meetings, etc. It is a behavior that indirectly contributes to the personal safety of workers and encourages the development of a supportive work environment. Safe participation includes various activities, including assisting in resolving safety-related issues, actively participating in voluntary safety activities, and attending safety conferences. The key to safety participation is to help colleagues promote safety plans in the workplace, showcase initiative, and strive to improve workplace safety. Put another way, safety participation illustrates behaviors that do not directly affect the physical safety of employees, but can help the public understand the environment that supports safety (Neal et al., 2000). In this context, safety participation is seen as a form of situational expression.

### 2.3 Integrating relationships between leadership and safety performance

Recently, a significant amount of research has quantified the relationship between leadership styles and safety performance from different perspectives. Overall, most of them are based on accident theory and social exchange theory perspectives. Accident theory suggests that accidents or injuries caused by employees working in risk workplaces are the result of management failures, and safety failures can also be attributed to leadership styles (Arifin et al., 2025; Beatriz et al., 2017; Neal et al., 2000; Payne et al., 2025; Suwaidi et al., 2025; Ta et al., 2022; Liu Y. Y. et al., 2022). Social exchange theory provides insight into these relationships. The theory suggests that employee behavior in the workplace is based on social exchange, and relationships between members should follow the principle of reciprocity, meaning that both parties in the exchange should benefit or at least not suffer losses. Under this theory, the leader-employee relationships within an organization are influenced by positive behavior, and employees will do things beneficial to their leaders and the organization based on the principle of reciprocity (Cropanzano, 2005). To build a strong integrative theory, rather than treating the 21 examined leadership styles in isolation, we organize our theoretical framework around the underlying mechanisms that link these behaviors to varying safety outcomes. While individual leadership labels remain conceptually distinct, they frequently operate through shared behavioral pathways. It should be noted that these pathways act as theoretical heuristics rather than strictly mutually exclusive categories, as many complex leadership styles inherently span multiple mechanisms. Broadly, we categorize the impact of leadership on safety performance into four core theoretical pathways: (1) The Control and Monitoring Pathway: This pathway primarily drives safety compliance through structural oversight, rule enforcement, and explicit expectations. Leadership styles such as Transactional, Authoritarian, Autocratic, and Supervisor leadership operate heavily through this mechanism. By emphasizing task completion, unilateral decision-making, and performance-based exchange (Beatriz et al., 2014; Kapp, 2012; Wang and Gong, 2017), leaders ensure that employees adhere strictly to standardized safety procedures. While effective for short-term compliance, extreme reliance on strict control (e.g., authoritarian or autocratic styles) may inadvertently inhibit employees’ proactive safety participation and initiative due to a lack of autonomy (Saleem et al., 2021). (2) The Motivation and Meaning Pathway: This pathway fosters proactive safety behaviors by instilling intrinsic motivation, shared vision, and personal meaning in safety goals. Styles such as Transformational, Spiritual, Empowering, Authentic, Positive, and Safety leadership exemplify this mechanism. Leaders utilizing this pathway inspire subordinates through personal charisma, moral examples, and transparent communication, actively promoting safety as a core cultural value (Ali et al., 2020; Avolio and Gardner, 2005; Gong et al., 2023; Gracia et al., 2020; Inness et al., 2010; Quansah et al., 2023; Shen and Ding, 2022). By making safety personally meaningful and encouraging independent decision-making, this pathway strongly stimulates voluntary safety participation. (3) The Relational Support Pathway: Rooted heavily in social exchange theory, this pathway enhances overall safety outcomes through mutual trust, inclusivity, and a deep focus on subordinate well-being. This pathway encompasses Leader-member exchange (LMX), Ethical, Servant, Inclusive, and Paternalistic leadership. By prioritizing employees’ personal needs, establishing high-quality interpersonal exchanges, and ensuring all members feel respected and valued (Brondino et al., 2020; He et al., 2021; Liu P. et al., 2022; Shafique et al., 2020; Wang and Dong, 2023), leaders trigger a reciprocal obligation. Consequently, employees are motivated to both comply with safety regulations and actively participate in safety initiatives to benefit the collective. (4) The Depleting and Destructive Pathway: Conversely, negative leadership styles undermine safety performance by depleting employees’ cognitive and emotional resources. Styles such as Abusive, Destructive, Narcissistic, Passive, Laissez-faire, and negative Humor leadership fall into this category. Hostile, self-centered, or mockingly aggressive behaviors create anxiety and tension, signaling to employees that their physical well-being is not a priority (Li and Li, 2021; Wang and Gong, 2017; Wang et al., 2017; Wei, 2023). Similarly, passive and laissez-faire leaders fail to provide necessary guidance, creating dangerous role ambiguity (Jiang and Probst, 2016). Ultimately, this pathway inherently disrupts both task-oriented safety compliance and relational safety participation. Based on the above, we hypothesis that:

*H1a-H1o*: Leader-member exchange, Authentic leadership, Authoritarian leadership, Autocratic leadership, Empowering leadership, Ethical leadership, Safety leadership, Servant leadership, Spiritual leadership, Transactional leadership, Transformational leadership, Supervisor leadership, Paternalistic leadership, Inclusive leadership, Positive leadership are positively related to safety performance (including safety compliance or safety participation).

*H2a-H2f*: Abusive leadership, Destructive leadership, Laissez-faire leadership, Humor leadership, Narcissistic leadership, Passive leadership are negatively related to safety performance (including safety compliance or safety participation).

### 2.4 Moderators

#### 2.4.1 Cultural factors

National development level: Regardless of the leadership style is used, the national development level must be considered (Hofstede, 1980; Laskovaia et al., 2017; Rosenberg and Drucker, 1999; Li et al., 2026; Zhao et al., 2026). Because cultural, economic, and other environmental differences inherently shape safety performance paradigms. For example, highly productive countries with strong, politically stable and predictable legal frameworks for employee protection, and relatively mature institutionalization. Organizations and leaders are more active in efforts to reduce the risk of workplace and employee health. Therefore, lower risk behaviors and higher safety performance may lead developing countries. Institutionalization in developing countries is in need of further improvement, and reducing workplace risks is still faced with problems. In the complex and volatile high-risk working environment, it is particularly important for leaders to improve safety performance. In this study, national development level was coded according to the World Bank national income classification standard: samples from high-income economies were classified as the developed country group, and samples from upper-middle-income, lower-middle-income and low-income economies were classified as the developing country group (World Bank, 2025).

#### 2.4.2 Power factor

Power distance (PD): This is the extent to which an individual accepts and expects from power and hierarchy in organizations. It is the degree to which members with less power in society or organizations accept and expect unequal distribution of power, reflecting the extent to which power differentials are tolerated in interpersonal relationships. Power distance is one of the cultural parts of leadership behavior. National culture defines the values of employees and represents whether employees think they are equal to others and their expectations for leadership behavior, which affects some leadership behaviors (Dickson et al., 2012; House, 1971). Implicit leadership theory suggests that individuals from a wide range of cultural groups might have different understandings of the meaning of leadership. Cultures with high power distance tend to attach importance to hierarchy, authority, and obedience to superiors, while cultures with low power distance emphasize equality, participatory decision-making, and open communication between individuals and authoritative figures. Each of these internal leadership archetypes varies across high and low power distance cultures (Den Hartog et al., 1999). For example, lower PD cultures have more egalitarian values: employees prefer their managers to prioritize individual choices, flexibility, and personal ownership. In cultures with lower power distance, the leader is expected to offer socio-emotional support and to reduce reliance on authority and power. In a culture with high power distance, by contrast, employees honor formal authorities, anticipate that leaders will mentor them and exert power, regard their leaders as parents or elders, and are more at risk of tolerating abuse (Hon and Lu, 2016). Given the power differences between leaders and employees, power distance is particularly important in understanding the impact of leadership on employees’ attention and behaviors. For coding operation, we adopted national-level power distance scores from the Hofstede Culture Dimension Index (Hofstede et al., 2010), and took the global norm median of 50 as the classification threshold. Samples from countries with a score ≥ 50 were categorized as the high power distance group, and those with a score < 50 were categorized as the low power distance group, which is consistent with standard practice in cross-cultural meta-analyses (Li et al., 2020; Rockstuhl et al., 2020).

#### 2.4.3 Methodological factors

The extent of the influence of leadership style on safety performance may also vary according to the characteristics of the study (Hollenbeck and Wright, 2017). This study concerned 4 methodological moderators: publication time, measurement method, research design and sample collection method. (1) Publication time: The more recent the publication date, the deeper the understanding of the constructs is likely to be. Organizations and leaders are more likely to take some measures to decrease workplace risk and consider the health of employees, so the regulatory effect may be more significant; (2) Measurement method: The use of multilevel regression analysis versus structural equation modeling may reveal different aspects of the relationship. Multilevel regression may focus more on the analysis of the individual level, while structural equation modeling may consider the relationship between variables more comprehensively; (3) Research design: Experimental designs may provide more direct causal evidence through controlling variables, while field investigations may provide more complexity and reality close to the actual working environment; (4) Sample collection method: Data collected online may be easier to obtain large-scale samples, but may lack the depth and authenticity of face-to-face communication, while the data collected offline may be deeper, but the sample size may be limited. All methodological moderators were coded directly based on explicit descriptions in the method section of each original study, including publication year, analytical technique, research design and data collection mode.

Risk factors: Uncertainty avoidance refers to the assessment of an individual’s tolerance for fuzziness, uncertainty, and risk in all aspects of life (Kang and Kim, 2018; Watts et al., 2020). It reflects the degree to which members of society or organizations feel the threat of vague or uncertain situations, and seeks to minimize risks through rules, regulations and structured practices. In the organizational structure, employees who avoid high uncertainty tend to pay attention to stability, predictability and compliance with norms to reduce the psychological pressure and anxiety caused by uncertainty, which may consume employees’ cognitive resources and have a negative effect on employees’ safety performance and well-being (Fischer et al., 2019); while employees with low uncertainty avoidance may prefer a flexible working environment and hold a more open and tolerant attitude towards ambiguity and innovation, believing that these factors can promote personal growth and organizational performance. When formulating a vision, some leaders (such as transformational leadership) will show a positive image of the future and provide direction and meaning for employees, thereby reducing anxiety and uncertainty (Strange and Mumford, 2002). Consistent with the coding of power distance, uncertainty avoidance was coded using national-level scores from the Hofstede Culture Dimension Index (Hofstede et al., 2010), with the global median score of 50 as the cut-off point. Samples from countries with a score ≥ 50 were assigned to the high uncertainty avoidance group, and those with a score < 50 were assigned to the low group.

Occupational risk level is a key factor affecting leadership behavior and safety performance in a specific industry. The inherent risk level of an industry may change the effectiveness of leaders in workplace safety and employees’ work behavior (Willis et al., 2017), and in the risk workplace environment, the possibility of employees’ physical injury at work is higher than that in the normal environment, there is ambiguity in the trade-off between this and the goal of safety performance. In situations where safety performance is essential, workers might hold different aspirations for their managers (Neal et al., 2000; Willis et al., 2017). When the risk level is high, for example, employees want their leaders to give clarity and decrease ambiguity, such as in high-risk industries like construction, mining, oil and gas industry. Therefore, leadership that focuses on tasks and provides clear direction may be more effective (Watts et al., 2020). Echoing the debate, employees may have less tolerance for passive leadership behaviors within high-risk industries, which are characterized by a lack of leadership goals and actions. In low-risk occupations, leaders also need to pay attention to the safety of employees, but may pay more attention to preventive measures and continuous improvement. For example, in the office environment, leaders can pay attention to the details of employees’ sitting posture, display height and keyboard position, so as to reduce the risk of occupational diseases. In addition, leaders can also improve employees’ safety awareness and ability to deal with emergencies by regularly organizing safety training and drills. For coding purposes, we classified occupational risk levels with reference to the guidelines on Occupational Safety and Health (OSH) risk management, risky workplaces is defined as environments exposing employees to measurable physical, chemical, biological, or severe operational hazards (Andersen et al., 2019; Dogbla et al., 2023; International Labor Organization, 2024), based on the industry and workplace characteristics reported in each original study. The specific classification criteria are as follows: (1) High risk: workplaces with significant physical, chemical or nuclear hazards, high accident incidence and high fatality risk, including mining, construction, hazardous chemical manufacturing, nuclear power, oil and gas extraction, and civil aviation; (2) Medium risk: workplaces with moderate occupational health risks and medium accident incidence, including general manufacturing, ground public transportation, healthcare, and warehousing logistics; (3) Low risk: workplaces dominated by indoor office work with extremely low accident incidence, including finance, education, administrative services, and information technology. Two coders independently classified each sample, and all discrepancies were resolved through group discussion.

## 3 Method

### 3.1 Literature search

**Figure 2:**
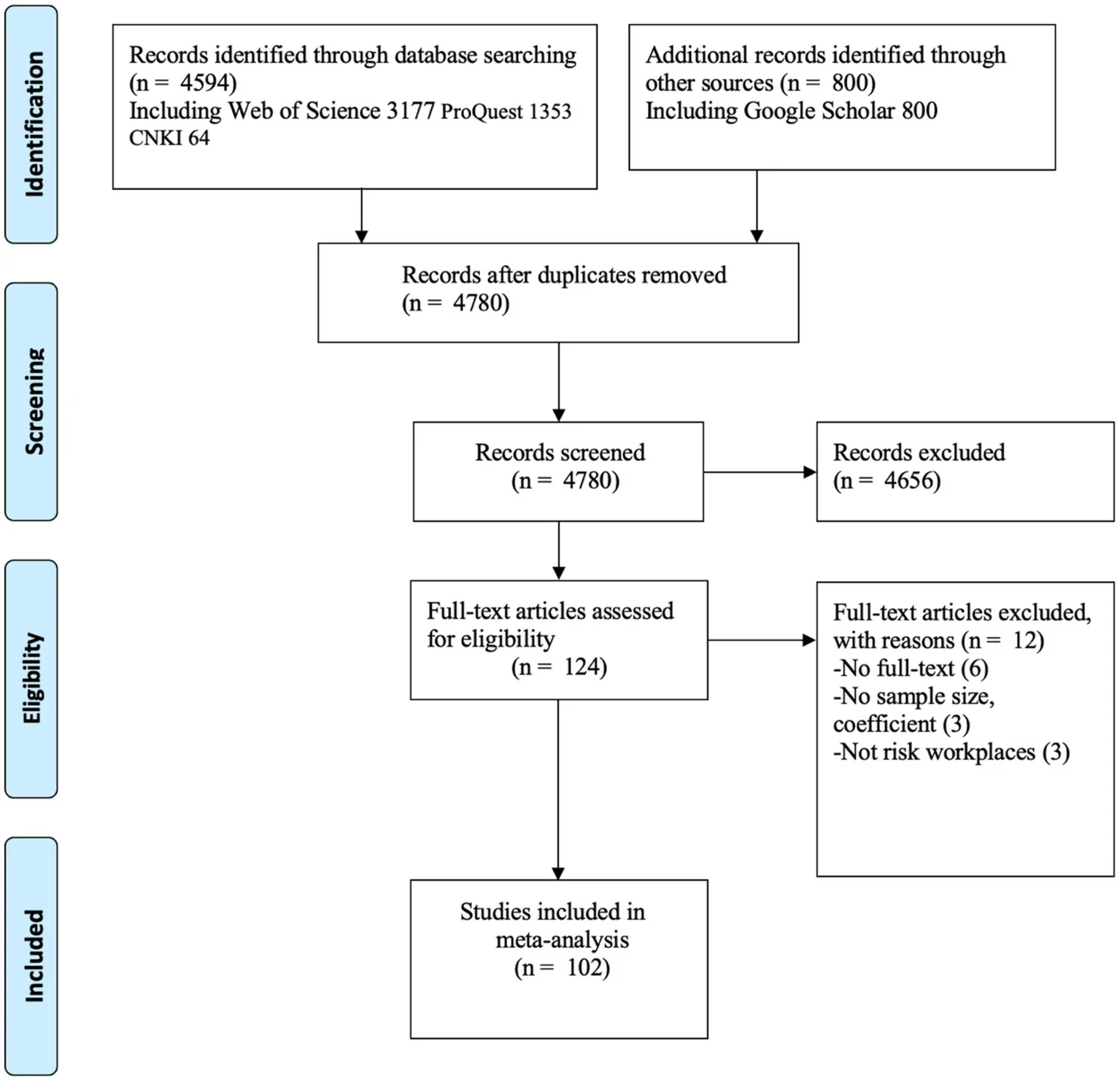
The PRISMA diagram for search and selection process.

A systematic search was conducted to capture relevant literature under the guidelines of information retrieval experts. First, we searched the following international electronic databases to identify potentially relevant published and unpublished research: Google Scholar, Web of Science, Campbell Library, CNKI (China National Knowledge Infrastructure), and ProQuest (Dissertations & Theses) (until December 1st, 2025) (Barends and Rousseau, 2018; Li et al., 2020; Li et al., 2025). The systematic search was performed by combining each of the possible combinations of three sets of keywords: (1) leadership-related keywords (e.g., “leadership*,” “leader,” “supervisor*,” “director*,” “manager*,” “CEO*,” “administrator*,” and “executive*”); (2) safety-related keywords (e.g., “securi*,” “safe*,” “injur*,” “accident*,” and “hazard*”); (3) performance (e.g., “performance*,” “participation*,” “compliance*,” “effectiveness,” “achievement*,” “merit*,” and “efficacy”). This method is consistent with previous meta-analyses or bibliometrics (Li et al., 2026; Siddaway et al., 2019). Second, to minimize the impact of publication bias and capture potentially interesting literature as fully as possible, we have also included unpublished conference papers, research plans, and theses in the aforementioned database. Finally, we used the snowball method to review and consult the reference sections of the acquired literature to identify other potential sources. Overall, we reviewed a total of 5,394 studies in order to include those that met the criteria in the meta-analysis. The search process is shown in Figure 2.

### 3.2 Inclusion criteria

The following four criteria were utilized to identify studies for inclusion in the meta-analysis: First, they should empirically examine any associations (leadership and safety performance) of interest. Second, the study should report sufficient data to calculate the effect size (e.g., sample size and correlation coefficient) of at least one relationship considered in this study. Third, to extend our findings to safety and risk management, we explicitly operationalized ‘risky workplaces’ as occupational environments where employees face measurable physical, chemical, biological, or severe operational hazards that directly threaten occupational health and safety. While our literature search spanned databases across the fields of health, safety, environment, business, management, political science and public administration, studies were only retained if the sample data were drawn from relevant operational contexts. We primarily targeted samples from employees and managers working at high and medium risk jobs (such as construction site workers, pilots, highway public service providers, nuclear material processors and so on). Samples from lower-risk environments (e.g., general office or administrative settings) were only included if they explicitly measured safety compliance and participation (e.g., ergonomic hazard mitigation or emergency drill participation), specifically to serve as a comparative baseline for our occupational risk moderator analyses. Fourth, considering the potential differences in research designs and variable measurement methods, we only included studies that measured leadership and safety performance using self-reporting. This decision was made to rigorously preserve construct measurement consistency across the meta-analytic sample. Mixing highly disparate operationalizations of safety performance—such as subjective perceptual scales, supervisor-rated performance, safety observations, and objective archival incident or injury records—can introduce severe methodological heterogeneity and distort the synthesized effect sizes. Furthermore, an initial screening of the literature revealed an overwhelming preponderance of self-report measures regarding safety behaviors in this specific domain, making it the most viable standard for ensuring a comprehensive and methodologically homogenous comparison.

### 3.3 Coding process

To verify coding accuracy, we established a coding team of three researchers with knowledge and methodological experience in safety, risk and management. Coding was independently coded by two researchers of our team for each included study. First, the two researchers reached an agreement on coding criteria via intensive discussions and began the first round of operational coding, covering potentially numerous research features that we believed may be related to later analysis. These included authors, publication time, region, sample size, population, collected types, design, methods, leadership styles, safety performance and so on. Second, based on the above, the two coders independently encoded all relevant research features of the included studies. Third, the two cross-checked and verified their code consistency (reached 90%) and then merged these into the final code table. In the implementation coding, all issues happened were discussed by the team, and the final coding table was determined. Prior to formal coding, we developed a standardized coding manual that specified operational definitions, data sources, classification rules, decision trees and illustrative examples for all variables, including all moderating variables examined in this study, to ensure the consistency and replicability of coding.

The coding protocols for core moderating variables are detailed as follows:

Cultural and national-level moderators (national development level, power distance, uncertainty avoidance): These variables were coded based on publicly available national-level authoritative index data, rather than subjective judgment. National development level was classified according to the World Bank national income classification standard; power distance and uncertainty avoidance were coded using scores from the Hofstede National Culture Dimension Index (Hofstede et al., 2010), with the global norm median of 50 as the uniform cut-off point for binary classification.

Occupational risk level: Coded based on the industry and workplace type reported in each original study, with reference to the guidelines on Occupational Safety and Health (OSH) risk management. Three risk levels (high, medium, low) were defined with clear operational criteria and typical industry examples, as described in Section 2.4.

Methodological moderators: Coded directly based on the explicit descriptions in the method section of original studies, including publication year, analytical method (HLM vs. SEM), research design (field study vs. experiment) and data collection type (offline vs. online).

### 3.4 Effect size computing

Most of our studies are based on reported correlation coefficients, which examine the relationship between leadership styles and safety performance. The correlation coefficient is usually marked as *R* or *r*. However, other coefficients [such as R^2^, d, or standard deviation (SD)] have also been used in some studies. In this case, it is necessary to convert these coefficients into r, as follows (Card, 2011).

$$d=\frac{m1-m2}{s}$$

$$r=\frac{d}{\sqrt{4+d}}$$

where m1 and m2 are the means of Groups 1 and 2, respectively, and s is the pooled SD across the two groups.

$$r=\frac{{\left(1-R^{2}\right)}^{2}}{n-1}$$

$R^{2}$ was the determinant coefficient, and n was the sample size.

$$Z_{i}=0.5\ln(\frac{1+r_{i}}{r_{i}}),SE_{i}=\frac{1}{\sqrt{W_{i}}},Z=\frac{\sum (W_{i}*Z_{i})}{\sum W_{i}},r=\frac{e^{2z}-1}{e^{2z}+1.}$$

$W_{i}$ = n – 3, where Z is the Z-score when calculating Fisher’s Z.

### 3.5 Relative weight analyses

In the real world, the correlation between variables is common, and the problem of collinearity of variables faces difficulties. Relative weight analysis is one of the methods to solve this problem. It can accurately separate the individual contributions of each variable. Therefore, we use this method to estimate the relative importance of different leadership styles on safety performance (Johnson and LeBreton, 2004), to find out what leadership style had the greatest impact on safety performance. Relative weight analysis computes the epsilon weight of each predictor variable as the ratio of overall explanatory variance, and permits comparisons to be made between predictor variables based on this ratio. We use RWA package to calculate the original epsilons of the independent variable and the relative weights after rescaling (i.e., explained variance percentage).

### 3.6 Risk of bias assessment

Two authors evaluated the included studies’ risk of bias, and two researchers independently extracted the data. It is a measurement tool often used to evaluate the quality of studies. This tool is adapted from the bias risk tool previously developed for observational studies, which represents the degree to which the deviation between systematic error and true values affects the research results. It has seven items, and with “yes,” “no,” or “unclear” options for each item.

### 3.7 Moderating effect analysts

There are many separate empirical studies in meta-analysis, and resemblance is a requirement for conducting moderator analysis. Weighted merging could handle multiple isomorphic statistics. Through the Q value analysis of 102 empirical studies, it is greater than the corresponding chi-square test value, indicating that the samples’ effect sizes for this study is heterogeneous. In addition, the values of I^2^ are mostly higher than 75% (considered high heterogeneity in meta-analysis standards, according to Higgins and Thompson's (2002) study, I^2^ values of 75, 50 and 25% reflect high, medium and low heterogeneity, respectively). For example, the value of I^2^ is 95.4%, indicating that the difference of actual effect size accounts for 95.4% of the observed change. Random error accounts for 4.6% of the observed variance. Hence, we could conclude that there are moderators in the selected sample literature. Many potential factors may lead to inconsistencies in previous investigations of leadership styles and safety performance. These factors include publication time, cultural background (such as national development level in different geographical regions), methods, power distance, occupational risk level and other factors (Li et al., 2020; Hunter and Schmidt, 2004).

### 3.8 Meta-analytic procedures

In order to make the results more realistic and reliable, we used Schmidt and Hunter’s (2004) random effects model for the analysis; Microsoft’s Excel 2021 (version 16.92) to extract and organize the data; R (version 4.2.3)‘s “metafor” package to perform meta-analysis, moderators’ analysis and publication bias analysis, etc. First, the correlation coefficients reported in each study and the internal consistency reliability coefficient were used to calculate the real correlation coefficient between leadership and safety performance (r also known as the zero-order correlation coefficient, which is the correlation coefficient after the weighted observation of the average sample size or the real correlation coefficient after measurement error correction). Among them, for a few studies that reported other effect size (such as d, mean and standard deviation), we converted them into a unified comprehensive effect size r. We have corrected the estimated values of measurement and sampling errors. Internal consistency reliability often overestimates the reliability of score-based standards (which in turn underestimates the correlation of corrections), while alpha correction reduces the magnitude of attenuation effects, as internal measurement errors are typically smaller than inter measurement errors (Lebreton and Senter, 2008). For measures without reported reliability coefficients, we used an interpolation method based on the average reliability of the corresponding measures in the encoded dataset. To make the estimated effect size more accurate, we calculated the 95% confidence interval (CI) around each effect. We also calculated a 90% confidence interval (CV) as an indicator of generalization level; Wide confidence intervals indicate the presence of moderating factors, while zero crossing intervals indicate incomplete generalizability of the results (Hunter and Schmidt, 2004). To rigorously address the issue of statistical dependence among multiple effect sizes drawn from the same study, we applied distinct procedures depending on the level of analysis. For the overall meta-analysis, for studies that included multiple measures and/or outcome measures, we calculated the composite r for inclusion to ensure that each unique study contributed only a single aggregate effect size, thereby strictly preserving the assumption of independence. Conversely, for the leadership-style-specific and moderator analyses, the unit of analysis was shifted to the specific relationship level. In these instances, k refers to the number of independent samples or individual effect sizes rather than the number of unique studies, which explains why the cumulative k in subgroup tables frequently exceeds the total study count of 102. When a single study contributed multiple effect sizes corresponding to completely distinct constructs (e.g., reporting separate correlations for both transformational and transactional leadership), these effect sizes were treated as separate data points in their respective construct-specific categories. To appropriately handle the within-study dependence generated by retaining multiple effect sizes from the same study in these sub-analyses, we applied robust variance estimation (RVE) within our random-effects modeling framework. Next, to better assess the impact of the publication bias profile of the included studies, we used a combination of Fail-safe N (Rosenthal) and Egger’s regression because both methods have advantages and disadvantages for testing different samples. For example, Egger regression is prone to Type I error for small sample sizes and, therefore, Egger regression is not applicable to small Fail-safe N (Rosenthal). Their combination compensates for this shortcoming by reflecting the required number of studies to turn around the results of a meta-analysis, and will measure how potentially unpublished number of studies with negative results affects the results of positive meta-analyses (Li et al., 2020; Schaeuffele et al., 2024). It is difficult to shift the results of a meta-analysis when Fail-safe N is much larger than the number of included studies, especially when the number of studies exceeds 5 K + 10, where K is the number of included studies (Card, 2011). However, given that their judgment criteria are not the golden rule, we combined them to assess the impact of the included studies owing to publication bias.

## 4 Results

### 4.1 Sample characteristics

We finally selected 102 articles from 5,394 articles as a sample, and 107,109 participants from 24 countries on five continents participated in these studies. The time span was 1999–2024. Among them, most of the participants were from developing countries (63.7%), 59.8% of the samples were from Asia, followed by Europe (23.5%) and North America (10%). Most of these participants work in medium and high-risk workplaces, such as mining sites, construction sites, hazardous chemicals, nuclear power, aviation manufacturing and transportation. Notably, in the nearly 15 years since the study was published, there has been increasing interest in these relationships. About 90% of all the included articles were published after 2010, especially about 60% of the articles were published in the past 5 years, and the number of published articles showed a significant growth trend.

### 4.2 Meta-analysis results of leadership styles and safety performance

The results of meta-analysis are shown in Table 1. Overall, among the 21 leadership styles examined, 17 were significantly related to safety performance, and most of them had a true effect value of 0.3 or higher, indicating that leadership had a significant and important impact on safety performance (i.e., from moderate to severe).

**Table 1:** Bivariate relationships between leadership styles and safety performance.

Leadership styles	Safety performance types	K	N	r	SD	CI 95%	CV 80%	I^2^	F-N
Overall	Safety performance	102	107,109	0.22	0.12	[0.17; 0.22]	[0.08; 0.36]	95.4	18111***
	Safety compliance	106	56,300	0.2	0.1	[0.17; 0.23]	[0.07; 0.33]	92.1	13642***
	Safety participation	161	50,809	0.19	0.11	[0.17; 0.21]	[0.05; 0.33]	86.1	12573***
Leader-member exchange	Safety performance	16	5,749	0.24	0.06	[0.14; 0.33]	[0.16; 0.32]	93.2	1069***
	Safety compliance	6	1938	0.21	0.06	[0.07; 0.35]	[0.13; 0.29]	94.5	118***
	Safety participation	6	2,804	0.29	0.04	[0.12; 0.44]	[0.24; 0.34]	95.5	380***
Abusive leadership	Safety performance	6	2065	−0.16	0.05	[−0.47; 0.19]	[−0.22; −0.10]	98.3	45
	Safety compliance	2	794	0.35	0.05	[0.14; 0.53]	[0.29; 0.41]	90.3	***
	Safety participation	2	794	−0.27	0.05	[−0.37; −0.16]	[−0.33; −0.21]	63.8	***
Authentic leadership	Safety performance	14	5,237	0.37	0.19	[0.29; 0.44]	[0.13; 0.61]	90.4	2775***
	Safety compliance	2	5,237	0.28	0.19	[0.22; 0.34]	[0.26; 0.52]	0	***
	Safety participation	4	1996	0.37	0.12	[0.27; 0.46]	[0.22; 0.52]	83.9	309***
Authoritarian leadership	Safety performance	4	1704	0.05	0.13	[−0.06; 0.15]	[−0.12; 0.22]	62.3	0
	Safety compliance	-	-	-	-	-	-	-	-
	Safety participation	-	-	-	-	-	-	-	-
Autocratic leadership	Safety performance	4	1,588	0.04	0.28	[−0.24; 0.31]	[−0.40; 0.32]	97	0
	Safety compliance	2	794	0.26	0.2	[0.01; 0.49]	[0.00; 0.52]	93	*
	Safety participation	2	794	−0.19	0.13	[−0.33; −01]	[−0.36; −0.02]	79.1	*
Destructive leadership	Safety performance	9	2,841	−0.33	0.05	[−0.54; −0.08]	[−0.41; −0.29]	97.4	449***
	Safety compliance	3	948	0.54	0.24	[0.41; 0.66]	[0.23; 0.85]	86.8	****
	Safety participation	3	948	−0.39	0.07	[−0.45; −0.34]	[−0.48; −0.30]	0	****
Empowering leadership	Safety performance	15	3,914	0.25	0.12	[0.19; 0.31]	[0.10; 0.40]	75.4	1039***
	Safety compliance	7	1849	0.23	0.16	[0.13; 0.32]	[0.02; 0.44]	79.5	185***
	Safety participation	8	2065	0.28	0.15	[0.20; 0.35]	[0.09; 0.47]	72.8	339***
Ethical leadership	Safety performance	18	8,479	0.24	0.14	[0.14; 0.32]	[0.06; 0.42]	94.7	2315***
	Safety compliance	3	1,413	0.24	0.09	[0.19; 0.29]	[0.12; 0.36]	0	58***
	Safety participation	6	2,330	0.23	0.16	[0.05; 0.40]	[0.02; 0.44]	95	241***
Laissez-faire leadership	Safety performance	4	1,588	−0.01	0.2	[−0.37; 0.36]	[−0.27; 0.25]	98.3	-
	Safety compliance	1	397	−0.48	-	-	-	-	-
	Safety participation	2	794	0.27	-	[0.07; 0.46]	-	89.2	**
Safety leadership	Safety performance	34	18,869	0.22	0.16	[0.17; 0.27]	[0.01; 0.43]	92.6	6155***
	Safety compliance	14	4,881	0.17	0.12	[0.12; 0.22]	[0.02; 0.32]	85.6	984***
	Safety participation	7	2,124	0.16	0.06	[0.02; 0.30]	[0.08; 0.24]	91.3	150*
Servant leadership	Safety performance	10	3,667	0.25	0.18	[0.12; 0.36]	[0.02; 0.48]	93.3	548***
	Safety compliance	4	1,394	0.17	0.12	[0.02; 0.30]	[0.02; 0.32]	86.1	31*
	Safety participation	4	1,394	0.26	0.19	[0.08; 0.43]	[0.02; 0.50]	91.7	92***
Spiritual leadership	Safety performance	32	17,678	0.17	0.11	[0.13; 0.20]	[0.03; 0.31]	82.4	4107***
	Safety compliance	14	7,594	0.18	0.12	[0.12; 0.24]	[0.03; 0.33]	84.9	905***
	Safety participation	14	7,594	0.15	0.08	[0.01; 0.19]	[0.05; 0.25]	71.8	572***
Transactional leadership	Safety performance	17	4,358	0.19	0.1	[0.09; 0.28]	[0.06; 0.32]	91.2	683***
	Safety compliance	8	1949	0.17	0.12	[0.03; 0.31]	[0.02; 0.32]	90.3	114*
	Safety participation	6	1,573	0.21	0.14	[0.11; 0.31]	[0.03; 0.43]	77.3	106***
Transformational leadership	Safety performance	82	21,543	0.24	0.14	[0.21; 0.28]	[0.06; 0.42]	87.7	16627***
	Safety compliance	38	9,612	0.2	0.1	[0.14; 0.25]	[0.07; 0.33]	88.2	3964***
	Safety participation	36	9,717	0.28	0.19	[0.22; 0.33]	[0.04; 0.52]	88	6523***
Supervisor leadership	Safety performance	1	402	0.35	-	-	-	-	***
	Safety compliance	1	402	0.35	-	-	-	-	***
	Safety participation	-	-	-	-	-	-	-	-
Paternalistic leadership	Safety performance	1	305	0.761	-	-	-	-	***
	Safety compliance	-	-	-	-	-	-	-	-
	Safety participation	-	-	-	-	-	-	-	-
Humor leadership	Safety performance	1	397	−0.305	-	-	-	-	***
	Safety compliance	-	-	-	-	-	-	-	-
	Safety participation	-	-	-	-	-	-	-	-
Inclusive leadership	Safety performance	2	918	0.47	0.09	[0.37; 0.57]	[0.35; 0.59]	73	***
	Safety compliance	-	-	-	-	-	-	-	-
	Safety participation	-	-	-	-	-	-	-	-
Narcissistic leadership	Safety performance	2	474	−0.18	0.08	[−0.27; −0.09]	[−0.28; −0.08]	0	***
	Safety compliance	-	-	-	-	-	-	-	-
	Safety participation	-	-	-	-	-	-	-	-
Passive leadership	Safety performance	1	169	−0.3	-	-	-	-	***
	Safety compliance	1	169	−0.3	-	-	-	-	***
	Safety participation	-	-	-	-	-	-	-	-
Positive leadership	Safety performance	1	316	0.134	-	-	-	-	***
	Safety compliance	-	-	-	-	-	-	-	-
	Safety participation	-	-	-	-	-	-	-	-

There must be at least three rows of data to run publication bias procedures and there must be some variance in the standard errors, *p ≤ 0.01, **p ≤ 0.005, ***p ≤ 0.001. Additionally, k indicates the number of independent effect sizes; N indicates the cumulative sample size; r represents the comprehensive correlation coefficient; SD represents the standard deviation; CI 95% represents the 95% confidence interval; CV 80% represents the 80% credibility interval; and F-N represents fail-safe N.

On the correlation between different leadership styles and safety performance, overall, the comprehensive effect value of leadership styles and safety performance is 0.22 (k = 102, n = 107,109). Specifically, Leader-member exchange (r = 0.24), Authentic leadership (r = 0.37), Empowering leadership (r = 0.25), Ethical leadership (r = 0.24), Safety leadership (r = 0.22), Servant leadership (r = 0.25), Spiritual leadership (r = 0.17), Transactional leadership (r = 0.19), Transformational leadership (r = 0.24), Supervisor leadership (r = 0.35, k = 1), Paternalistic leadership (r = 0.76, k = 1), Inclusive leadership (r = 0.47, k = 2), and Positive leadership (r = 0.13, k = 1) are significantly positively correlated with safety performance (the confidence interval does not include zero). Therefore, hypotheses H1a, H1b, h1e, H1f, h1g, h1h, h1i, H1J, H1k, h1l, h1m, H1n and H1o are supported.

Conversely, Destructive leadership (r = −0.33), Humor leadership (r = −0.31, k = 1), Narcissistic leadership (r = −0.18, k = 2), and Passive leadership (r = −0.30, k = 1) were significantly negatively correlated with safety performance (the confidence interval does not include zero). Therefore, hypotheses H2b, H2d, H2e and h2f are supported.

However, Authoritarian leadership (r = 0.05), Autocratic leadership (r = 0.04) Abusive leadership (r = −0.16) and Laissez-faire leadership (r = −0.01) were not significantly related to safety performance (the confidence interval includes zero). Therefore, hypotheses H1c, H1d, H2a and H2c are not supported.

### 4.3 Relative weight analysis

We analyzed the relative contribution of each leadership type to safety performance. Table 2 shows the meta-analytic correlation matrix of leadership variables. Although relative weight analyses did not have a smallest number of effect sizes required for every coefficient, we summarized the leadership and safety performance correlation coefficients with significant and combined effect values, number of included studies, and total sample size, we used normal guidelines to exclude leadership categories with less than 2 effect sizes (Barends and Rousseau, 2018; Li et al., 2020; Valentine et al., 2010). Therefore, we conducted a relative weight analysis based on 10 leadership types (namely, Leader-member exchange, authentic leadership, destructive leadership, empowering leadership, ethical leadership, safety leadership, servant leadership, spiritual leadership, transactional leadership and transformational leadership). The results show that transformational leadership has greater relative importance (21.42%) in explaining the safety performance of risk workplaces, followed by authentic leadership (21.27%) and Leader-member exchange (11.08%), while other leadership styles such as moral leadership, safety leadership, and transactional leadership are shown in Table 3.

**Table 2:** The meta-analytic correction matrix of the relationship between leadership style and safety performance.

	LMX	AUL	DEL	EML	ETL	SAL	SEL	SPL	TAL	TFL
LMX	1									
AUL	0.701, 7/1633	1								
DEL	0.62, 2/708	0.17, 3/300	1							
EML	0.47, 6/2133	0.71, 6/2335	−0.27, 2/486	1						
ETL	0.71, 18/4052	0.45, 4/578	−0.10, 2/500	0.46, 7/2066	1					
SAL	0.62, 2/474	0.33, 7/3108	−0.21, 6/1106	0.40, 6/1809	0.58, 6/1892	1				
SEL	0.67, 8/2382	0.67, 7/3346	−0.31, 2/450	0.45, 6/1020	0.40, 6/1042	0.34, 6/1422	1			
SPL	0.45, 6/2005	0.12, 2/400	−0.25, 2/400	0.55, 6/678	0.38, 7/1036	0.24, 8/2190	0.36, 6/900	1		
TAL	0.50, 6/1879	0.40, 4/770	−0.27, 9/1510	0.47, 6/900	0.69, 13/2232	0.20, 6/1289	0.45, 6/920	0.35, 6/890	1	
TFL	0.73, 20/5451	0.75, 10/2397	−0.41, 8/1405	0.35, 6/1000	0.70, 20/3717	0.23, 6/1752	0.52, 5/774	0.44, 6/720	0.65, 152/56789	1

LMX, Leader-member exchange, AUL, Authentic leadership, DEL, Destructive leadership, EML, Empowering leadership, ETL, Ethical leadership, SAL, Safety leadership, SEL, Servant leadership, SPL, Spiritual leadership, TAL, Transactional leadership, TFL, Transformational leadership. The comprehensive correlation coefficient is shown in the table, k indicates the number of independent effect sizes, N is the cumulative sample sizes.

**Table 3:** The relative importance of different leadership styles in predicting safety performance.

Variables	RW	%R^2^
Leader-member exchange	0.11	11.08
Authentic leadership	0.21	21.27
Destructive leadership	0.05	5.41
Empowering leadership	0.08	8.08
Ethical leadership	0.08	7.96
Safety leadership	0.09	8.55
Servant leadership	0.03	2.85
Spiritual leadership	0.05	4.81
Transactional leadership	0.09	8.58
Transformational leadership	0.21	21.42

RW represents the relative weight (the epsilon weight of each predictor variable); %R^2^ represents the percentage of overall explanatory variance attributed to the specific leadership style.

### 4.4 Moderators’ test

As shown in Table 1, the I^2^ value of all test results is higher than 75%, and their heterogeneity Q test is significant. Therefore, it can be considered that the correlation is not homogeneous, and there may be moderators affecting their correlation. As the proposed research model, we further studied the possible boundary conditions of the correlations [i.e., cultural factors (national development level), power factors (power distance), methodological factors (publication time, measurement method, research design and sample collection method), risk factors (uncertainty avoidance and occupational risk level)].

In terms of cultural factors, the national development level significantly moderates the relationship between 10 styles of leadership and safety performance (Table 4). Overall, the impact of developed countries on the relationships between them is slightly stronger than that of developing countries (0.20 vs. 0.19). It is worth noting that the relationships is also affected by national development level. For example, developing countries’ effect sizes for Leader-member exchange, authentic leadership, empowering leadership, ethical leadership, safety leadership, and transactional leadership on safety performance are stronger than those in developed countries, and both are greater than 0.30 (with moderating effect); developed countries, on the other hand, have a stronger relationship between transformational leadership and safety performance than developing countries (0.26 vs. 0.24).

**Table 4:** Meta-analysis results for leadership styles and safety performance: the role of national development level.

Moderators	Relationships	Models	Level	*k*	Point estimate	95%CI		Z	Q	Q_within	Q_between	*P*
National development level	Overall	Fixed	Developed	103	0.21	0.20	0.22	39.07	999.13	6167.9	2.69	0.000
		Fixed	Developing	181	0.20	0.19	0.20	53.62	5168.77			0.000
	Leader-member exchange-SP	Fixed	Developed	5	0.08	0.03	0.13	2.95	13.46	162.90	59.85	0.003
		Fixed	Developing	11	0.31	0.28	0.34	20.94	149.44			0.000
	Authentic leadership-SP	Fixed	Developed	2	0.25	0.14	0.34	5.00	0.54	127.12	7.77	0.000
		Fixed	Developing	12	0.39	0.36	0.41	28.45	126.58			0.000
	Destructive leadership-SP	Fixed	Developed	9	−0.33	−0.54	−0.08	−15.80	228.81	228.81	0.00	0.000
		Fixed	Developing	-	-	-	-	-	-			-
	Empowering leadership-SP	Fixed	Developed	13	0.24	0.21	0.28	13.96	34.57	34.81	22.02	0.000
		Fixed	Developing	2	0.42	0.35	0.47	11.97	0.24			0.000
	Ethical leadership-SP	Fixed	Developed	2	0.08	0.02	0.14	2.75	69.10	282.97	42.46	0.006
		Fixed	Developing	16	0.28	0.26	0.30	24.71	213.86			0.000
	Safety leadership-SP	Fixed	Developed	10	0.15	0.13	0.16	15.29	41.94	378.78	68.40	0.000
		Fixed	Developing	24	0.26	0.24	0.28	24.00	336.84			0.000
	Servant leadership-SP	Fixed	Developed	-	-	-	-	-	-	135.98	0.00	-
		Fixed	Developing	10	0.25	0.12	0.36	14.54	135.98			0.000
	Spiritual leadership-SP	Fixed	Developed	-	-	-	-	-	-	176.13	0.00	
		Fixed	Developing	32	0.17	0.13	0.20	22.29	176.13			0.000
	Transactional leadership-SP	Fixed	Developed	8	0.17	0.11	0.22	6.18	36.14	177.44	2.74	0.000
		Fixed	Developing	9	0.22	0.18	0.25	11.91	141.30			0.000
	Transformational leadership-SP	Fixed	Developed	59	0.26	0.24	0.27	31.05	578.74	649.45	1.40	0.000
		Fixed	Developing	22	0.24	0.22	0.26	20.89	70.71			0.000

SP, Safety performance. Z represents the Z-value for the significance test; Q represents the total heterogeneity; k indicates the number of independent effect sizes; Q_within represents the heterogeneity within groups; Q_between represents the heterogeneity between groups. *p ≤ 0.01, **p ≤ 0.005, ***p ≤ 0.001.

Among the power factors (Table 5), on the whole, high power distance has a stronger significant positive moderating effect on the relationship than low power distance (0.21 vs. 0.18). Among them, compared with low power distance, high power distance has a stronger significant positive moderating effect on the relationship between nine leadership styles and safety performance except destructive leadership. It is worth noting that high power distance has a significant negative moderating effect on the relationship between destructive leadership and safety performance (it is −0.33).

**Table 5:** Meta-analysis results for leadership styles and safety performance: The role of power distance.

Moderators	Relationships	Models	Level	*k*	Point estimate	95%CI		Z	Q	Q_within	Q_between	P
Power distance	Overall	Fixed	High	191	0.21	0.21	0.22	58.33	4368.01	6140.04	30.55	0.000
		Fixed	Low	93	0.18	0.17	0.19	32.03	1772.03			0.000
	Leader-member exchange-SP	Fixed	High	10	0.32	0.30	0.35	21.57	121.89	138.81	83.93	0.000
		Fixed	Low	6	0.06	0.01	0.11	2.47	16.93			0.010
	Authentic leadership-SP	Fixed	High	13	0.38	0.36	0.41	28.70	129.14	129.14	5.75	0.000
		Fixed	Low	1	0.20	0.05	0.35	2.56	0.00			0.010
	Destructive leadership-SP	Fixed	High	9	−0.33	−0.54	−0.08	−15.80	228.81	228.81	0.00	0.000
		Fixed	Low	-	-	-	-	-	-			-
	Empowering leadership-SP	Fixed	High	15	0.25	0.19	0.31	17.78	56.83	56.83	0.00	0.000
		Fixed	Low	-	-	-	-	-	-			0.000
	Ethical leadership-SP	Fixed	High	9	0.3	0.27	0.32	22.38	134.59	296.92	28.51	0.000
		Fixed	Low	9	0.18	0.15	0.22	10.17	162.33			0.000
	Safety leadership-SP	Fixed	High	25	0.25	0.23	0.27	22.37	307.94	409.33	37.85	0.000
		Fixed	Low	9	0.16	0.14	0.18	16.70	101.39			0.000
	Servant leadership-SP	Fixed	High	10	0.25	0.12	0.36	14.54	135.98	135.98	0.00	0.000
		Fixed	Low	-	-	-	-	-	-			-
	Spiritual leadership-SP	Fixed	High	32	0.17	0.13	0.20	22.29	176.13	176.13	0.00	0.000
		Fixed	Low	-	-	-	-	-	-			-
	Transactional leadership-SP	Fixed	High	12	0.26	0.23	0.29	15.55	89.58	114.42	65.77	0.000
		Fixed	Low	5	−0.04	−0.11	0.03	−1.18	24.84			0.24
	Transformational leadership-SP	Fixed	High	32	0.26	0.24	0.28	25.83	133.68	649.35	1.5	0.000
		Fixed	Low	49	0.24	0.23	0.26	27.08	515.67			0.000

SP, Safety performance. Z represents the Z-value for the significance test; Q represents the total heterogeneity; k indicates the number of independent effect sizes; Q_within represents the heterogeneity within groups; Q_between represents the heterogeneity between groups. *p ≤ 0.01, **p ≤ 0.005, ***p ≤ 0.001.

In terms of methodological factors, publication time had no significant impact on the relationships (Table 6). To address our research questions regarding methodological boundary conditions, Table 7 presents the moderating effects of different measurement methods—specifically comparing the use of Hierarchical Linear Modeling (HLM) and Structural Equation Modeling (SEM). Overall, the measurement method significantly moderated the relationship between leadership styles and safety performance (Q_between = 116.85, *p* < 0.001). Studies utilizing SEM yielded a significantly stronger overall effect size (0.22) compared to those using HLM (0.15). For example, the estimated relationships for leader member exchange, authentic leadership, ethical leadership, safety leadership and servant leadership were stronger when analyzed via SEM rather than HLM. In some other relationships, however, the measurement method based on HLM yielded a stronger effect size than the measurement method based on SEM (for example, destructive leadership, empowering leadership, transactional leadership, and transformational leadership). The research design has different moderating effects on the relationship between different leadership styles and safety performance (Table 8). For example, the field approach has a stronger moderating effect than the experimental approach (0.21vs 0.15), safety leadership, and transactional leadership. It is worth noting that the experimental approach is more effective than the field approach in the relationship between transformational leadership and safety performance (0.27 vs. 0.8). In terms of sample collection methods, in the impact of some leadership styles on safety performance, offline collection of samples has a stronger moderating effect than online collection (Table 9, for example, overall (0.22 vs. 0.14), safety leadership, transactional leadership and transformational leadership), while in some leadership styles, online collection of samples has a stronger moderating effect than offline collection (for example, authentic leadership, destructive leadership and ethical leadership).

**Table 6:** Meta-analysis results for leadership styles and safety performance: the role of publication time.

Moderators	Relationships	Models	*k*	Point estimate	95%CI		Q	Z	R^2^	*P*
Publication time	Overall	Regression	102	0.00	−0.01	0.01	0.29	−0.54	0.00	0.587
	Leader-member exchange-SP	Regression	16	0.00	0.00	0.00	3.05	1.75	0.23	0.08
	Authentic leadership-SP	Regression	14	0.03	0.00	0.06	3.48	1.86	0.21	0.060
	Destructive leadership-SP	Regression	9	0.06	−0.20	0.05	1.52	−1.23	0.11	0.220
	Empowering leadership-SP	Regression	15	−0.01	−0.03	0.02	0.41	−0.64	0.00	0.520
	Ethical leadership-SP	Regression	18	0.03	0.00	0.07	3.44	1.86	0.09	0.060
	Safety leadership-SP	Regression	34	0.00	−0.01	0.02	0.26	0.51	0.00	0.610
	Servant leadership-SP	Regression	10	0.04	−0.04	0.10	0.74	0.86	0.04	0.390
	Spiritual leadership-SP	Regression	32	0.00	−0.02	0.03	0.11	0.33	0.00	0.740
	Transactional leadership-SP	Regression	17	−0.04	−0.09	0.00	3.37	−1.84	0.14	0.070
	Transformational leadership-SP	Regression	82	0.01	0.00	0.02	1.59	1.26	0.00	0.210

SP, Safety performance. Z represents the Z-value for the significance test; Q represents the total heterogeneity; k indicates the number of independent effect sizes; Q_within represents the heterogeneity within groups; Q_between represents the heterogeneity between groups. *p ≤ 0.01, **p ≤ 0.005, ***p ≤ 0.001.

**Table 7:** Meta-analysis results for leadership styles and safety performance: the role of measure method.

Moderators	Relationships	Models	Level	*k*	Point estimate	95%CI		Z	Q	Q_within	Q_between	*P*
Measure method	Overall	Fixed	HLM	79	0.15	0.13	0.16	23.67	2430.09	6053.74	116.85	0.000
		Fixed	SEM	205	0.22	0.21	0.23	62.89	3623.66			0.000
	Leader-member exchange-SP	Fixed	HLM	3	0.07	−0.03	0.16	1.39	9.80	205.04	17.71	0.160
		Fixed	SEM	13	0.27	0.25	0.30	20.08	195.24			0.000
	Authentic leadership-SP	Fixed	HLM	5	0.29	0.25	0.33	12.80	6.46	105.56	29.32	0.000
		Fixed	SEM	9	0.43	0.40	0.45	26.23	99.10			0.000
	Destructive leadership-SP	Fixed	HLM	3	−0.48	−0.52	−0.43	−15.86	5.43	175.41	53.40	0.000
		Fixed	SEM	6	−0.20	−0.25	−0.15	−7.17	169.98			0.000
	Empowering leadership-SP	Fixed	HLM	1	0.32	0.24	0.40	7.24	0.00	55.70	1.13	0.000
		Fixed	SEM	14	0.27	0.24	0.30	16.28	55.70			0.000
	Ethical leadership-SP	Fixed	HLM	7	0.21	0.17	0.24	12.74	43.69	306.14	19.3	0.000
		Fixed	SEM	11	0.30	0.27	0.32	20.80	262.44			0.000
	Safety leadership-SP	Fixed	HLM	1	0.11	0.00	0.22	1.90	0.00	444.82	2.35	0.057
		Fixed	SEM	33	0.20	0.18	0.21	27.20	444.82			0.000
	Servant leadership-SP	Fixed	HLM	1	0.13	0.04	0.23	2.77	0.00	130.42	5.57	0.006
		Fixed	SEM	9	0.25	0.22	0.28	14.47	130.42			0.000
	Spiritual leadership-SP	Fixed	HLM	-	-	-	-	-	-	176.13	0.00	-
		Fixed	SEM	32	0.17	0.13	0.20	22.29	176.13			0.000
	Transactional leadership-SP	Fixed	HLM	7	0.27	0.22	0.31	12.16	26.23	161.53	18.65	0.000
		Fixed	SEM	10	0.14	0.10	0.18	6.94	135.31			0.000
	Transformational leadership-SP	Fixed	HLM	24	0.26	0.24	0.28	21.80	202.64	649.54	1.31	0.000
		Fixed	SEM	57	0.25	0.23	0.26	30.41	446.90			0.000

SP, Safety performance. Z represents the Z-value for the significance test; Q represents the total heterogeneity; k indicates the number of independent effect sizes; Q_within represents the heterogeneity within groups; Q_between represents the heterogeneity between groups. *p ≤ 0.01, **p ≤ 0.005, ***p ≤ 0.001.

**Table 8:** Meta-analysis results for leadership styles and safety performance: the role of design.

Moderators	Relationships	Models	Level	*k*	Point estimate	95%CI		Z	Q	Q_within	Q_between	*P*
Design	Overall	Fixed	Field	256	0.21	0.20	0.22	64.22	5936.4	6126.03	44.56	0.000
		Fixed	Experiment	28	0.15	0.13	0.17	17.86	189.66			0.000
	Leader-member exchange-SP	Fixed	Field	16	0.26	0.23	0.28	19.68	222.75	222.75	0.00	0.000
		Fixed	Experiment	-	-	-	-	-	-			-
	Authentic leadership-SP	Fixed	Field	14	0.37	0.29	0.44	28.69	134.89	134.89	0.00	0.000
		Fixed	Experiment	-	-	-	-	-	-			-
	Destructive leadership-SP	Fixed	Field	9	−0.33	−0.54	−0.08	−15.80	228.81	228.81	0.00	0.000
		Fixed	Experiment	-	-	-	-	-	-			-
	Empowering leadership-SP	Fixed	Field	15	0.25	0.19	0.31	17.78	56.83	56.83	0.00	0.000
		Fixed	Experiment	-	-	-	-	-	-			0.000
	Ethical leadership-SP	Fixed	Field	18	0.24	0.14	0.32	24	325.43	325.43	0.00	0.000
		Fixed	Experiment	-	-	-	-	-	262.44			-
	Safety leadership-SP	Fixed	Field	28	0.26	0.24	0.28	24.94	351.72	372.32	74.86	0.000
		Fixed	Experiment	6	0.14	0.12	0.16	13.94	21.00			0.000
	Servant leadership-SP	Fixed	Field	10	0.25	0.12	0.36	14.54	135.98	135.98	0.00	0.000
		Fixed	Experiment	-	-	-	-	-	-			-
	Spiritual leadership-SP	Fixed	Field	32	0.17	0.13	0.20	22.29	176.13	176.13	0.00	0.000
		Fixed	Experiment	-	-	-	-	-	-			-
	Transactional leadership-SP	Fixed	Field	14	0.21	0.18	0.24	2.92	178.56	179.06	1.12	0.000
		Fixed	Experiment	3	0.15	0.05	0.25	13.03	0.5			0.003
	Transformational leadership-SP	Fixed	Field	20	0.18	0.15	0.21	11.18	163.77	626.19	24.66	0.000
		Fixed	Experiment	61	0.27	0.25	0.28	36.04	462.42			0.000

SP, Safety performance. Z represents the Z-value for the significance test; Q represents the total heterogeneity; k indicates the number of independent effect sizes; Q_within represents the heterogeneity within groups; Q_between represents the heterogeneity between groups. *p ≤ 0.01, **p ≤ 0.005, ***p ≤ 0.001.

**Table 9:** Meta-analysis results for leadership styles and safety performance: the role of collected types.

Moderators	Relationships	Models	Level	*k*	Point estimate	95%CI		Z	Q	Q_within	Q_between	*P*
Collected types	Overall	Fixed	Offline	233	0.22	0.22	0.23	63.82	4122.24	5969.86	200.74	0.000
		Fixed	Online	51	0.14	0.12	0.15	20.06	1100.68			0.000
	Leader-member exchange-SP	Fixed	Offline	16	0.26	0.23	0.28	19.68	222.75	222.75	0.00	0.000
		Fixed	Online	-	-	-	-	-	-			-
	Authentic leadership-SP	Fixed	Offline	4	0.31	0.26	0.35	12.53	2.91	119.55	15.34	0.000
		Fixed	Online	10	0.41	0.38	0.44	26.10	116.64			0.000
	Destructive leadership-SP	Fixed	Offline	6	−0.20	−0.25	−0.15	−7.17	169.98	175.41	53.40	0.000
		Fixed	Online	3	−0.48	−0.52	−0.43	−15.86	5.43			0.000
	Empowering leadership-SP	Fixed	Offline	15	0.25	0.19	0.31	17.78	56.83	56.83	0.00	0.000
		Fixed	Online	-	-	-	-	-	-			0.000
	Ethical leadership-SP	Fixed	Offline	3	0.05	0.00	0.11	1.95	178.34	254.64	70.80	0.000
		Fixed	Online	15	0.30	0.27	0.32	25.16	76.30			0.050
	Safety leadership-SP	Fixed	Offline	26	0.27	0.25	0.29	24.06	344.07	374.10	73.08	0.000
		Fixed	Online	8	0.15	0.13	0.16	15.35	30.04			0.000
	Servant leadership-SP	Fixed	Offline	10	0.25	0.12	0.36	14.54	135.98	135.98	0.00	0.000
		Fixed	Online	-	-	-	-	-	-			-
	Spiritual leadership-SP	Fixed	Offline	32	0.17	0.13	0.20	22.29	176.13	176.13	0.00	0.000
		Fixed	Online	-	-	-	-	-	-			-
	Transactional leadership-SP	Fixed	Offline	14	0.16	0.12	0.20	7.45	95.16	151.34	28.85	0.000
		Fixed	Online	3	0.08	0	0.16	2.01	37.67			0.04
	Transformational leadership-SP	Fixed	Offline	56	0.26	0.25	0.28	35.26	412.67	637.73	13.12	0.000
		Fixed	Online	25	0.2	0.17	0.23	13	225.05			0.000

SP, Safety performance. Z represents the Z-value for the significance test; Q represents the total heterogeneity; k indicates the number of independent effect sizes; Q_within represents the heterogeneity within groups; Q_between represents the heterogeneity between groups. *p ≤ 0.01, **p ≤ 0.005, ***p ≤ 0.001.

In terms of risk factors, uncertainty avoidance exhibited varying moderating effects on the relationship (Table 10). For example, in some leadership styles, low uncertainty avoidance has a stronger moderating effect than high uncertainty avoidance [such as overall (0.21 vs. 0.19), Leader-member exchange, safety leadership, and transactional leadership]. It is worth noting that in some leadership styles, high uncertainty avoidance has a stronger moderating effect than low uncertainty avoidance (for example, authentic leadership, ethical leadership, spiritual leadership and transformational leadership). It is interesting to note that occupational risk levels (high, medium and low) have a U-shaped moderating effect on most relationships (Table 11). For example, in these specific correlations, the moderating effects of high, medium and low are weakened and then enhanced in turn (for example, ethical leadership, safety leadership, servant leadership and transactional leadership). However, this pattern does not universally apply across all leadership archetypes; for instance, transformational leadership demonstrated a continuous positive linear trend from high to low risk.

**Table 10:** Meta-analysis results for leadership styles and safety performance: the role of uncertainty avoidance.

Moderators	Relationships	Models	Level	*k*	Point estimate	95%CI		Z	Q	Q_within	Q_between	*P*
Uncertainty Avoidance	Overall	Fixed	High	107	0.19	0.18	0.20	35.74	1501.26	6162.03	8.57	0.000
		Fixed	Low	177	0.21	0.20	0.21	55.94	4660.77			0.000
	Leader-member exchange-SP	Fixed	High	4	0.06	0.01	0.12	2.15	7.71	157.20	65.55	0.030
		Fixed	Low	12	0.31	0.28	0.33	21.17	149.48			0.000
	Authentic leadership-SP	Fixed	High	3	0.39	0.32	0.46	9.79	20.82	134.81	0.08	0.000
		Fixed	Low	11	0.38	0.35	0.40	26.96	114.00			0.000
	Destructive leadership-SP	Fixed	High	9	−0.33	−0.54	−0.08	−15.80	228.81	228.81	0.00	0.000
		Fixed	Low	-	-	-	-	-	-			-
	Empowering leadership-SP	Fixed	High	15	0.25	0.19	0.31	17.78	56.83	56.83	0.00	0.000
		Fixed	Low	-	-	-	-	-	-			0.000
	Ethical leadership-SP	Fixed	High	6	0.27	0.23	0.32	11.72	48.38	324.73	0.71	0.000
		Fixed	Low	12	0.25	0.23	0.27	20.95	276.34			0.000
	Safety leadership-SP	Fixed	High	12	0.14	0.11	0.18	7.55	108.40	437.43	9.75	0.000
		Fixed	Low	22	0.21	0.19	0.22	26.35	329.03			0.000
	Ethical leadership-SP	Fixed	High	-	-	-	-	-	-	135.98	0.00	-
		Fixed	Low	10	0.25	0.12	0.36	14.54	135.98			0.000
	Spiritual leadership-SP	Fixed	High	24	0.17	0.15	0.18	19.78	124.14	176.13	0.01	0.000
		Fixed	Low	8	0.16	0.14	0.20	10.28	51.99			0.000
	Transactional leadership-SP	Fixed	High	7	0.17	0.11	0.22	7.00	26.01	178	2.49	0.000
		Fixed	Low	10	0.22	0.18	0.25	11.89	151.69			0.000
	Transformational leadership-SP	Fixed	High	29	0.26	0.23	0.28	21.51	250.54	650.63	0.22	0.000
		Fixed	Low	52	0.25	0.23	0.26	30.60	400.09			0.000

SP, Safety performance. Z represents the Z-value for the significance test; Q represents the total heterogeneity; k indicates the number of independent effect sizes; Q_within represents the heterogeneity within groups; Q_between represents the heterogeneity between groups. *p ≤ 0.01, **p ≤ 0.005, ***p ≤ 0.001.

**Table 11:** Meta-analysis results for leadership styles and safety performance: the role of occupational risk level.

Moderators	Relationships	Models	Level	*k*	Point estimate	95%CI		Z	Q	Q_within	Q_between	*P*
Occupational risk level	Overall	Fixed	High	190	0.18	0.18	0.19	53.10	4368.22	6035.48	135.12	0.000
		Fixed	Medium	58	0.21	0.19	0.22	26.43	1222.85			0.000
		Fixed	Low	36	0.3	0.28	0.32	31.43	444.41			0.000
	Leader-member exchange-SP	Fixed	High	14	0.26	0.24	0.28	19.59	215.61	219.92	2.82	0.000
		Fixed	Medium	2	0.16	0.04	0.27	2.56	4.32			0.010
		Fixed	Low	0	-	-	-	-	-			-
	Authentic leadership-SP	Fixed	High	12	0.35	0.33	0.38	23.98	67.75	118.33	16.56	0.000
		Fixed	Medium	2	0.47	0.42	0.52	16.25	50.58			0.010
		Fixed	Low	-	-	-	-	-	-			-
	Destructive leadership-SP	Fixed	High	7	−0.24	−0.29	−0.19	−9.69	181.28	181.75	47.07	0.000
		Fixed	Medium	2	−0.52	−0.57	−0.46	−14.24	0.46			0.000
		Fixed	Low	-	-	-	-	-	-			-
	Empowering leadership-SP	Fixed	High	15	0.25	0.19	0.31	17.78	56.83	56.83	0.00	0.000
		Fixed	Medium	-	-	-	-	-	-			0.000
		Fixed	Low	-	-	-	-	-	-			-
	Ethical leadership-SP	Fixed	High	14	0.25	0.22	0.27	21.05	281.43	281.75	43.68	0.000
		Fixed	Medium	3	0.23	0.18	0.29	8.18	0.32			0.000
		Fixed	Low	1	0.6	0.51	0.68	10.44	0			0.000
	Safety leadership-SP	Fixed	High	23	0.19	0.18	0.21	24.99	311.12	410.35	36.83	0.000
		Fixed	Medium	7	0.11	0.04	0.17	11.94	51			0.000
		Fixed	Low	4	0.0.34	0.29	0.39	3.35	49.13			0.001
	Servant leadership-SP	Fixed	High	7	0.33	0.29	0.37	16.41	69.13	69.14	66.84	0.000
		Fixed	Medium	2	0.04	−0.03	0.1	1.19	0.02			0.006
		Fixed	Low	1	0.13	0.04	0.23	2.77	0			0.234
	Spiritual leadership-SP	Fixed	High	24	0.17	0.15	0.18	19.78	124.14	176.13	0.01	0.000
		Fixed	Medium	8	0.17	0.14	0.2	10.28	51.99			0.000
		Fixed	Low	-	-	-	-	-	-			-
	Transactional leadership-SP	Fixed	High	11	0.21	0.18	0.24	12.02	143.41	178.92	1.26	0.000
		Fixed	Medium	5	0.17	0.11	0.23	5.46	35.51			0.000
		Fixed	Low	1	0.2	0.01	0.38	2.07	0			0.039
	Transformational leadership-SP	Fixed	High	30	0.22	0.2	0.24	19.67	162.91	634.12	16.74	0.000
		Fixed	Medium	23	0.26	0.23	0.28	25.68	156.57			0.000
		Fixed	Low	28	0.28	0.26	0.3	19.22	314.63			0.000

SP, Safety performance. Z represents the Z-value for the significance test; Q represents the total heterogeneity; k indicates the number of independent effect sizes; Q_within represents the heterogeneity within groups; Q_between represents the heterogeneity between groups. *p ≤ 0.01, **p ≤ 0.005, ***p ≤ 0.001.

### 4.5 Publication bias analysis

**Figure 3:**
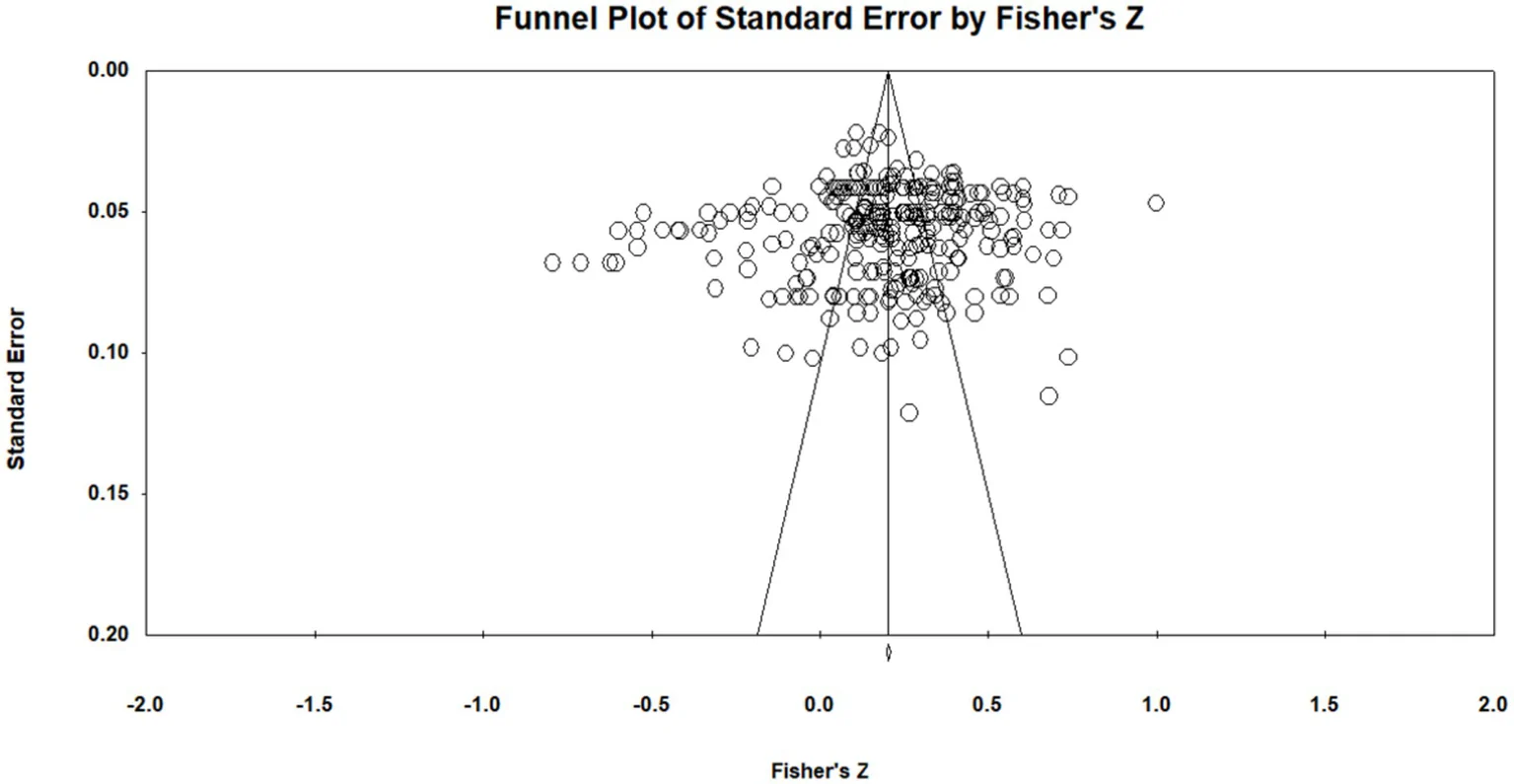
The funnel plot of included articles.

Publication bias (i.e., selective reporting) refers to any situation that may cause systematic deviation of conclusions from the true results in the process of data collection, analysis, interpretation and publication, such as self-examination by authors in order to meet theoretical expectations, or journals tend to support statistically significant results. In order to alleviate this concern, we use funnel plot, F-N number and Egger’s regression test to test publication bias. These three methods are usually based on defining the effect size distribution of a given zero order relationship, and their combination can make up for the situation that the sample is too large or insufficient. Figure 3 shows the total funnel plot of included studies. It seems be funnel shaped, with mostly points clustered around the top of the funnel-shaped graph, spreading downward and distributed evenly on each side of the centerline, minimizes the effects of publication bias. In addition, the larger the F-N value, the more reliable the results of the study. It was calculated that all the F-N values were much larger than 5 K + 10 (see Table 1). For example, the F-N number of overall leadership and leader member exchange are 18,111 and 1,069, respectively, (far greater than 5 k + 10, where k is 102 and 16 respectively). The intercept of the overall Egger’s regression test was −0.68 (*p* = 0.47), and all other Egger regression tests revealed a p greater than 0.05, it indicates that no meaningful difference exists between the intercept term and zero. Therefore, this demonstrates that the findings are comparatively credible and not markedly influenced by publication bias.

### 4.6 Article quality evaluation results

The included articles’ assessment is shown in Table 12. It has four key sections: blind method, withdrawal description, assignment hiding, and random generation. After assessment by two independent authors, in general, low risk was 89.9%, high risk was 7.8%, and unknown risk was 3.1%. The results showed that 91 articles had a high mean score, 8 articles was low, and 3 articles was unclear, so it can be considered that the quality of the studies included was high and had a low risk.

**Table 12:** The results of risk of bias.

Risk-of-bias criteria	Description	Risk-n(%) high	Risk-n(%) low	Risk-n(%) unclear
Selection bias	Is there a random allocation of subjects in the experiment? Is there random sampling in the study?	6 (5.9)	91 (89.2)	5(4.9)
Performance bias	Are the conditions outside the experiment consistent? Do investigators and respondents know the purpose of the study?	10 (9.8)	90 (88.2)	2 (2.0)
Detection bias	Is the standard measurement tool the same for the experimental group and the control group?	2 (2.0)	96 (94.1)	3 (2.9)
Attrition bias	What is the proportion of valid questionnaires, is there a missing value in the answers, and are there explanations for the above?	20 (19.6)	86 (84.4)	5 (4.9)
Reporting bias	Are the results of the study reported truthfully (i.e., both positive and negative results are reported)?	4 (3.9)	95 (93.2)	3 (2.9)
Other bias	Is the basic information description of the sample complete, and are the reliability and validity of the measurement tool in the study reported?	6 (5.9)	94 (92.1)	1 (1.0)

## 5 Discussion

In light of the high cost of risk workplace behaviors and activities, it is essential to be aware of the direction, magnitude, relative importance, and boundary conditions of the links within leadership styles and safety performance. It is important to clarify that our synthesis is based on safety performance operationalized as self-reported behaviors, as objective safety metrics were excluded to preserve construct measurement consistency. While previous few reviews have explored isolated leadership facets, this study is the first to systematically integrate 21 distinct leadership behaviors in the risk workplace into a general term “safety performance,” providing a comprehensive comparative analysis of their relative efficacy. we systematically used meta-analysis to deepen our understanding of the correlations. By integrating the structure of the same or closely related leadership behaviors in the risk workplace into a general term “safety performance,” we systematically synthesized the existing empirical work (K = 102, N = 107,109). To provide robust estimates of the correlations, the results reveal the significant correlation between them, and point out the relative weight of them, expand the nomological network of leadership related to safety performance, and consider a variety of possible regulatory factors and their boundary conditions.

First of all, in terms of the relationships between them, the results show that among the 21 leadership styles examined, 17 are significantly related to safety performance, and most of them have a real comprehensive effect value of 0.3 or higher, indicating that leadership style has a significant and important impact on safety performance (i.e., from moderate to severe). Rather than evaluating these styles merely as isolated labels, our meta-analytic results provide strong support for understanding their impacts through underlying integrative mechanisms. Specifically, the data reveal that leadership influences safety performance through distinct theoretical pathways. First, leadership styles operating through a motivation and meaning pathway (such as transformational, authentic, empowering, and spiritual leadership) emerged as some of the strongest contributors to safety performance. By instilling intrinsic motivation and framing safety as a core shared value, these leaders effectively stimulate voluntary safety participation and innovation. Second, styles leveraging a relational support pathway (such as Leader-member exchange, ethical, servant, and inclusive leadership) consistently and positively relate to safety performance by establishing high-quality interpersonal exchanges and prioritizing subordinate well-being, which triggers reciprocal safety compliance. Third, leadership styles operating primarily through a control and monitoring pathway (such as transactional and supervisor leadership) also yielded positive effects; these styles emphasize structural oversight, clear expectations, and performance-based exchanges, which are particularly effective for driving strict safety compliance.

Similarly, the data provide robust evidence for a depleting and destructive pathway, wherein negative leadership behaviors (such as passive, abusive leadership and destructive leadership) will reduce safety performance by draining employees’ cognitive and emotional resources and creating dangerous role ambiguity. Conversely, findings regarding several other leadership styles must be classified as tentative. Some of the leadership styles have fewer samples (such as k < 2 for supervisor leadership, paternalistic leadership, positive leadership, humor leadership, and passive leadership). Because these high effect sizes may be idiosyncratic or moderator-specific, we explicitly recommend careful use and interpretation of these results. Researchers and practitioners must avoid overstating the generalizability of these specific styles until further primary research is conducted to substantiate these preliminary trends. Although all leadership behaviors are important, the relative importance of different leadership styles varies greatly in the variance of the interpretation of safety performance variables. In particular, transformational leadership and authentic leadership are the largest contributors in the safety performance structure, which may be related to the characteristics of these leadership styles that encourage innovation, open communication and personal development. This result is consistent with previous research work (for example, Payne et al., 2025; Suwaidi et al., 2025; Willis et al., 2017).

Secondly, on the impact of diversity in leadership styles, we found that the impact of correlation was different. Specifically, paternalistic leadership and inclusive leadership showed an extremely high positive correlation (r = 0.761 and r = 0.47), which may be related to the protection and inclusiveness emphasized by these leadership styles, thus enhancing the safety awareness and behavior of employees (Liu et al., 2022; Wang and Dong, 2023). However, due to the small number of samples (k < 2), they are not robust, so they need to be carefully explained. On the contrary, destructive leadership is significantly negatively correlated with safety performance. The possible reason is that these leadership styles may weaken employees’ sense of security and belonging, which may negatively impact safety performance (Arifin et al., 2025; Durman et al., 2026; Jiang and Probst, 2016; Piao and Hahn, 2025; Wang et al., 2017).

Finally, in exploring the moderators, we found that the moderators’ test results revealed some boundary conditions of the relationship between them. In terms of cultural factors, especially the level of national development level, it has a significant moderating effect on the relationships. The moderating effect of developed countries on the relationship is slightly stronger than that of developing countries, which may be related to the relatively perfect safety regulations and high safety awareness in developed countries (Hofstede et al., 2010). In terms of power factors, power distance also shows a moderating effect on the relationship. High power distance countries have a stronger significant positive moderating effect on the relationship, which may be related to the more centralized leadership authority in high power distance (Parkkinen, 2024; Payne et al., 2025). In terms of methodological factors, measurement methods and research design have different moderating effects on the relationship. SEM has a stronger moderating effect than multilevel regression, which may be because the SEM can more comprehensively capture the complex relationship between them (Wendorf, 2002). Beyond that, the method of sample collection also shows a moderating effect on the relationship. Offline collection has a stronger moderating effect than online collection, which may be related to the fact that offline methods can provide more direct and in-depth data (Payne et al., 2025; Podsakoff et al., 2012; Suwaidi et al., 2025), In terms of risk factors, we found that the degree of uncertainty avoidance has different moderating effects on the relationship. In some relationships between leadership styles and safety performance, high uncertainty avoidance is more effective than low uncertainty avoidance, while in others, on the contrary, this may be related to the characteristics and scenarios of leadership styles. It is interesting to note that occupational risk level (high, medium and low risk) has a U-shaped moderating effect on the relationship between certain specific leadership styles (e.g., ethical, safety, servant, and transactional leadership) and safety performance. For this particular subset of leadership styles, their effectiveness may be most pronounced at the extremes of the occupational risk continuum in an environment with high occupational risk, the salience of highly structured, compliance-driven, or deeply supportive leadership behaviors may be amplified to mitigate immediate physical hazards and ensure strict implementation of rules and regulations. In contrast, in the medium occupational risk environment, the impact of these specific leadership styles may be attenuated, because in this environment, employees may not be too nervous or too lax, and conventional management methods might suffice to maintain the basic level of safety. Conversely, in low-risk settings, the nuances of these supportive leadership styles might again become highly relevant for fostering proactive safety participation (e.g., mitigating ergonomic or psychosocial risks). Because these moderating patterns vary distinctly depending on the specific leadership archetype being employed, organizations must proactively assess their occupational risk profiles and implement tailored leadership development initiatives. By leveraging evidence-based selection processes, individualized coaching, ongoing training, and consistent feedback, organizations can build a leadership pipeline equipped to meet the safety needs in different situations, so as to maximize the promotion of safe production, reduce the possibility of accidents, and build an efficient and safe working environment (Northouse, 2015; Sharon and Clarke, 2012; Suwaidi et al., 2025).

### 5.1 Theoretical significance

This meta-analysis has several theoretical implications for future research on leadership style and safety performance. First, we integrate various overlapping structures in safety performance research, and examines the extent to how different leadership styles are related to this overall structure. The results emphasize the key role of leadership in safety performance. Previous studies exploring predictors of safety performance largely relied on employees’ personality traits, demographic characteristics, and some leadership (such as safety leadership) or situational factors (such as work environment and needs) (Northouse, 2015; Payne et al., 2025; Piao and Hahn, 2025; Sharon and Clarke, 2012; Suwaidi et al., 2025; Zhao et al., 2022). To the best of our knowledge, this study is the first systematic meta-analysis of the role and boundary conditions of different leadership styles in relation to safety performance. Therefore, our study expands the meta-analytic nomological network of their relationships.

Secondly, while acknowledging the distinctiveness of the different forms, conceptually related leadership styles (e.g., transformational and transactional leadership) influence safety performance through shared underlying mechanisms rooted in common frameworks, such as transformative leadership and transactional leadership. Because the process of leadership influencing these different types of safety performance is similar, and they also draw from the same theories (for example, social exchange theory and social learning theory). Our empirical results substantiate this reasoning. Specifically, given that various leadership styles demonstrate robust, albeit directionally divergent (positive or negative) associations with safety outcomes, it is conceptually highly valuable to integrate these distinct leadership constructs into a unified theoretical framework rather than examining them in isolation. Our results show that both positive and negative leadership styles are important for safety performance. The combination of some leadership styles (rather than an ‘ideal’ leadership style, such as emerging safety leadership and ethical leadership) may be more effective in some aspects of safety performance. While organizational intervention tends to focus on a type of leadership behavior (for example, transformational leadership; Payne et al., 2025), focusing on a single type of leadership behavior may underestimate the relationships between them, and overestimate the predictive ability of this type. Therefore, empirical research and theoretical models may benefit from considering several categories or combinations of leadership styles simultaneously and considering their relative importance to safety performance, rather than theorizing around a ‘best,’ ‘most familiar,’ or ‘worst’ leadership style. Moving from empirical findings to practical applications, it is important to clearly delineate our data-driven conclusions from broader, more speculative practical implications. While our results robustly support the overarching value of combining constructive leadership styles, recommendations regarding exactly how organizations should intervene remain necessarily nuanced. In addition, these findings have key practical implications because as resources dwindle or are depleted, organizations often have limited resources for leadership training and employee safety, and the question of how to better optimize the allocation of resources is becoming an issue for organizations, practically, rather than speculatively asserting that leaders can effortlessly adapt their styles to fit ideal combinations, organizations should leverage these insights to inform long-term, systemic interventions. This would help them maximize their limited resources and efforts for leadership development, improving the work environment for their employees, reducing risky workplaces, and improving safety performance.

Third, by organizing the conceptualization of leadership around underlying behavioral mechanisms, our findings suggest that different theoretical pathways exert distinct influences on safety outcomes. Leadership styles operating primarily through the control and monitoring pathway appear crucial for ensuring fundamental safety compliance, whereas styles grounded in the motivation and meaning or relational support pathways are particularly vital for driving broader, voluntary safety participation. Because these theoretical pathways are not mutually exclusive and often overlap within specific leadership styles (e.g., safety leadership leveraging both monitoring and relational support), future theoretical models should move beyond isolated leadership labels. Instead, researchers should conceptualize how organizations can optimally blend these underlying mechanisms and balance necessary safety monitoring with inspirational and supportive behaviors to achieve holistic safety governance.

Finally, our study contributes by investigating the boundary conditions of the association between different leadership styles and safety performance. In particular, we tested whether and how cultural factors, power factors, methodological factors and risk factors moderate the relationship between them. For example, drawing upon implicit leadership theory regarding cultural variations (Den Hartog et al., 1999; Payne et al., 2025; Piao and Hahn, 2025), our analysis revealed that the efficacy of transformational leadership in promoting safety performance is heavily contingent upon a nation’s developmental context. Specifically, we found that the positive impact of transformational leadership on safety performance is significantly stronger in developed countries compared to developing countries. A plausible explanation is that in highly developed nations which typically possess mature regulatory frameworks and robust organizational support structures that leaders are more strongly expected, and better equipped, to actively champion employee well-being and potential. Similarly, under the risk factor (occupational risk level), the inherent hazard level of a specific industry also has an important impact on the boundary conditions of some comprehensive leadership behaviors (such as ethical leadership and safety leadership). Across the spectrum of workplace risk, ethical leadership and safety leadership are more closely related to higher safety performance at the extreme ends of the continuum (i.e., high and low occupational risk). When the risk is at the medium level, employees from these moderate-risk industries seem to be less concerned about ethical leadership and safety leadership.

### 5.2 Limitations and directions

Although this study has made some contributions in theory and practice, there are still some limitations. First of all, in the included studies, we did not directly measure the cultural level, power distance and risk factors, but used country-level proxies such as Hofstede et al. (2010) scores and UN classifications to code, usually taking the critical value of 50 on the index scale as the basis for dividing into high and low. Though this technique is consistent with prior meta-analyses (Li et al., 2020; Rockstuhl et al., 2020; Tang and Li, 2026), future empirical research should consider more detailed measurement, such as capturing these cultural and power variables at the individual or organizational level rather than relying on national averages, or dividing into extremely high, high, medium, low and extremely low. In addition, measuring cultural factors at the national level by the level of national development may not fully reflect the individual differences in the recognition of these cultural factors. Secondly, our research shows that for some leadership types (i.e., transformational leadership, transactional leadership, abusive leadership, authentic leadership, etc.), their correlation with safety performance is more easily moderated by methodological factors. This inherently creates a tension within our study: while we deliberately restricted our inclusion criteria to self-reported measures to maintain construct measurement consistency and avoid mixing subjective and objective archival data, we must acknowledge the methodological trade-off of this decision. Specifically, our reliance on self-reports means that objective safety outcomes (such as actual accident rates and injury frequencies) and multi-source evaluations (such as supervisor-rated safety performance) were excluded. While this decision ensures measurement homogeneity, it is a notable limitation because objective outcomes and multi-source data are particularly critical in safety research for capturing actual workplace incidents. Consequently, our findings reflect self-reported behavioral tendencies and may not perfectly align with objective safety outcomes. In other words, data collected from a single source (usually in the form of self-assessment) shows a greater effect than data collected from multiple sources (such as followers and leaders) or objective incident records. This supports the view that researches using self-reported study designs may have biased results because they typically report larger effect sizes. Therefore, future studies should be cautious in interpreting findings that rely solely on a single source (i.e., self-report) and should attempt to use more robust and diverse methods (such as multi-source studies and objective safety incident records) to avoid common methodological biases and to adequately triangulate these findings. Third, to build a strong integrative theory, we conceptualized the effects of leadership styles through distinct underlying mechanisms (e.g., control and monitoring, motivation and meaning, and relational support pathways). It must be added, however, that these pathways are heuristic conceptualizations rather than strictly mutually exclusive categories, as many complex leadership styles inherently span multiple mechanisms (for instance, transformational leadership may simultaneously provide motivation and relational support). Future empirical research should attempt to isolate and measure these mediating mechanisms directly to determine their independent and interactive effects on safety outcomes. Finally, although we have captured 21 leadership behaviors, not all potential leadership types were included, and some leadership behaviors yielded unstable results due to the small number of samples (k < 6). We recommend that these results be interpreted and applied cautiously, and that future studies continue to update and supplement these findings.

## 6 Conclusion

We finally selected 102 articles from 5,394 articles as a sample, and 107,109 participants from 24 countries on five continents participated in these studies. The time span was 1999–2024. Among them, most of them are from developing countries (63.7%), and 59.8% of the samples are from Asia. Most of these participants work in medium and high-risk workplaces, such as mining sites, construction sites, hazardous chemicals, nuclear power, aviation manufacturing and transportation. Most of the articles (about 90%) have been published after 2010. In particular, about 60% of the articles have been published in the past 5 years, and the number of articles published has shown a significant growth trend. We construct a meta-analysis theoretical framework to explain the impact of different leadership styles on safety performance. The results show that 13 leadership styles are significantly positively correlated with safety performance, and 4 are significantly negatively correlated with safety performance. The relative weight analysis shows that transformational leadership has the most interpretable contribution to safety performance. In addition, the moderating variable analysis shows that in some cases, the relationship between leadership styles and safety performance is moderated by cultural factors, power factors, methodological factors and risk factors. This study is the first to combine and analyze previous studies on this topic and highlights the importance of different leadership styles in enhancing or inhibiting and corporate safety governance.

## Data Availability

The original contributions presented in the study are included in the article/Supplementary material, further inquiries can be directed to the corresponding authors.
